# Clinical Value of Prognosis Gene Expression Signatures in Colorectal Cancer: A Systematic Review

**DOI:** 10.1371/journal.pone.0048877

**Published:** 2012-11-07

**Authors:** Rebeca Sanz-Pamplona, Antoni Berenguer, David Cordero, Samantha Riccadonna, Xavier Solé, Marta Crous-Bou, Elisabet Guinó, Xavier Sanjuan, Sebastiano Biondo, Antonio Soriano, Giuseppe Jurman, Gabriel Capella, Cesare Furlanello, Victor Moreno

**Affiliations:** 1 Unit of Biomarkers and Susceptibility (UBS), Catalan Institute of Oncology (ICO), Bellvitge Biomedical Research Institute (IDIBELL), and CIBERESP, L’Hospitalet de Llobregat, Barcelona, Spain; 2 Predictive Models for Biomedicine & Environment (PMBE), Fondazione Bruno Kessler (FBK), Trento, Italy; 3 Pathology Service, University Hospital Bellvitge (HUB – IDIBELL), L’Hospitalet de Llobregat, Barcelona, Spain; 4 General and Digestive Surgery Service, University Hospital Bellvitge (HUB – IDIBELL), L’Hospitalet de Llobregat, Barcelona, Spain; 5 Department of Clinical Sciences, Faculty of Medicine, University of Barcelona (UB), Barcelona, Spain; 6 Gastroenterology Service, University Hospital Bellvitge (HUB – IDIBELL), L’Hospitalet de Llobregat, Barcelona, Spain; 7 Hereditary Cancer Program, Catalan Institute of Oncology (ICO - IDIBELL), L’Hospitalet de Llobregat, Barcelona, Spain; Peter MacCallum Cancer Centre, Australia

## Abstract

**Introduction:**

The traditional staging system is inadequate to identify those patients with stage II colorectal cancer (CRC) at high risk of recurrence or with stage III CRC at low risk. A number of gene expression signatures to predict CRC prognosis have been proposed, but none is routinely used in the clinic. The aim of this work was to assess the prediction ability and potential clinical usefulness of these signatures in a series of independent datasets.

**Methods:**

A literature review identified 31 gene expression signatures that used gene expression data to predict prognosis in CRC tissue. The search was based on the PubMed database and was restricted to papers published from January 2004 to December 2011. Eleven CRC gene expression datasets with outcome information were identified and downloaded from public repositories. Random Forest classifier was used to build predictors from the gene lists. Matthews correlation coefficient was chosen as a measure of classification accuracy and its associated p-value was used to assess association with prognosis. For clinical usefulness evaluation, positive and negative post-tests probabilities were computed in stage II and III samples.

**Results:**

Five gene signatures showed significant association with prognosis and provided reasonable prediction accuracy in their own training datasets. Nevertheless, all signatures showed low reproducibility in independent data. Stratified analyses by stage or microsatellite instability status showed significant association but limited discrimination ability, especially in stage II tumors. From a clinical perspective, the most predictive signatures showed a minor but significant improvement over the classical staging system.

**Conclusions:**

The published signatures show low prediction accuracy but moderate clinical usefulness. Although gene expression data may inform prognosis, better strategies for signature validation are needed to encourage their widespread use in the clinic.

## Introduction

Colorectal cancer (CRC) is the third most common cancer worldwide and the second leading cause of cancer death. During the last decades, incidence has been increasing, while mortality has slowly been decreasing [1]. A remarkable feature of CRC is the difference in prognosis of the early and late stages of the disease: stage I and II have moderate risk of relapse after surgical resection, whereas patients with stage III have a higher chance of recurrence [2]. Recognized clinical risk factors for recurrence are emergency presentation, poorly differentiated tumor, depth of tumor invasion, and adjacent organ involvement (T4) [3]–[5]. However, these factors are insufficient to identify those patients with stage II CRC at high risk of recurrence and posterior metastasis or those patients with stage III CRC at low risk [6], leading to potential under-treatment or over-treatment [3].

Colon cancer metastasis is a tightly regulated process that requires aberrations in gene expression allowing cancer cells to progress through various steps until they colonize a distant organ [7]. Probably the alterations necessary for recurrence are already present in the primary colon carcinoma, which should allow identifying prognostic signatures [8]–[10]. Gene-expression profiling-based assays have been successful as prognostic tool in breast cancer [11], [12]. However, no signature has been adopted in routine clinical practice in CRC despite a large number of gene expression profiling studies on prognosis have been performed.

The aim of this work was to test the predictive ability of these published signatures as prognostic markers in a significant number of independent datasets, in order to understand their strengths and weakness and identify if any of them can be used clinically to guide decisions about adjuvant therapy for patients with stage II or III CRC.

## Materials and Methods

### Published Gene Expression Signatures and Validation Datasets

**Figure 1:**
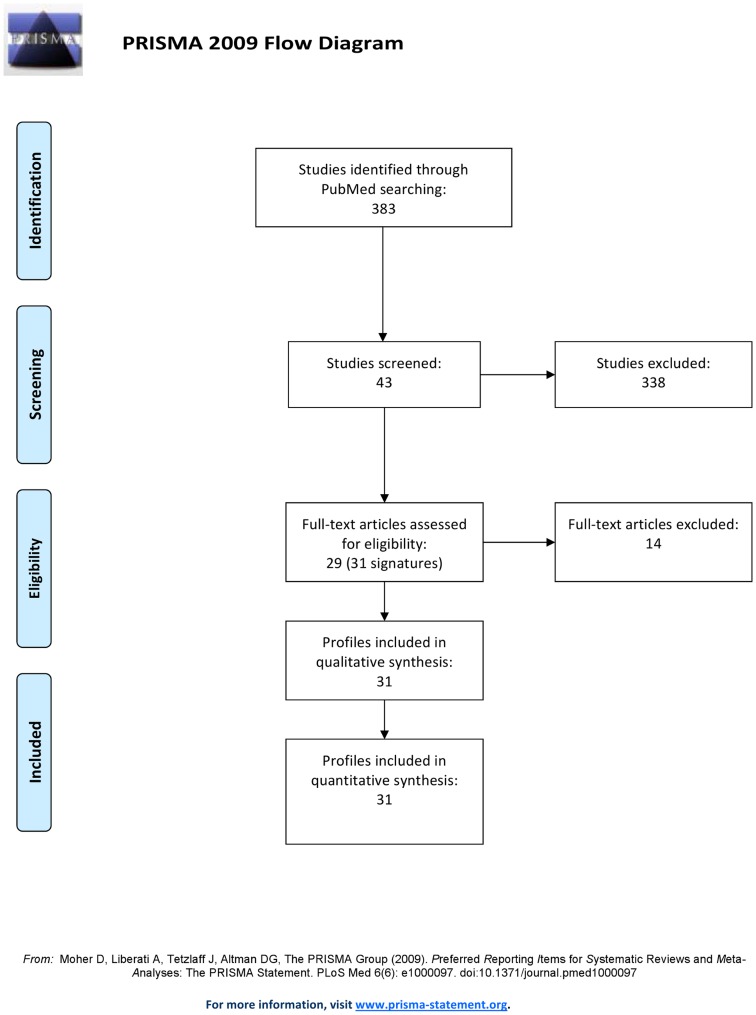
PRISMA Diagram which depicts the flow of information through the different phases of the prognosis signatures studies systematic review.

A systematic literature review was performed to identify studies that used gene expression data to predict prognosis in CRC [13]. The search was based on the PubMed database and was restricted to recent papers to increase validity (from January 2004 to December 2011). Files S1–S2, Table S1 and Figure 1 detail the selection protocol and PRISM checklist. Articles that provided a list of differentially expressed genes in primary tumor samples associated with CRC prognosis were included in our study. We indistinctly refer to these lists of genes as ‘signatures’ or ‘profiles’. Studies based on tissue microarray and those that exclusively were focused on differences between stages or between primary tumor and metastases were excluded. The studies finally included for analysis are described in Table 1 [14]–[43]. Publicly available datasets with whole-genome gene expression measures in CRC primary tumor samples were identified and downloaded from GEO [44] and ArrayExpress [45] microarray data repositories (Tables 2 and 3). Pre-processed series matrixes originally provided by the authors were used in our analysis.

**Table 1:** Description of signatures used in this work.

Signature	Signature size	Training sample size (good + poor)	Training outcome	Training platform	Signature validation	Independent validation outcome	Size of independent validation sample (good + poor)	Validation results	Reference
**AD09MSI**	13	65 (56+9)	Recurrence in colorectal tumor samples	Affymetrix	No validation	–	–	–	19156145 [14]
**AD09MSS**	3	195 (173+22)	Recurrence in colorectal tumor samples	Affymetrix	Independent expression dataset	Recurrence	50 (25+25)	Asociation	19156145 [14]
**AJ08**	36	20 (10+10)	A proliferation signature was derived from cell lines and a data set containing physiological expression of human colon crypts	MWG 30 K Oligo Set	Two independent expression datasets	Recurrence	108 (84+24)	Association	19238634 [15]
**AR05**	72	25 (15+10)	Recurrence in colorectal tumor samples	Affymetrix	Leave-one-out cv/Independent TMA (1 protein)	Recurrence	137*	88% accuracy/Association	16143127 [16]
**BD07**	8	16 (10+6)	Recurrence in colorectal tumor samples	Human 19 K Oligo Array	No validation	–	–	–	17390049 [17]
**BR05**	30	18 (9+9)	Recurrence in colorectal tumor samples	Affymetrix	3-fold cv	–	–	78% accuracy	16091735 [18]
**BR06**	30	50 (25+25)	Recurrence in colorectal tumor samples	Affymetrix	2-fold cv/k-fold Montecarlo cv	–	–	80% accuracy/76% accuracy	16966692 [19]
**BT04**	244	24 (13+9)	Metastasis in colorectal tumor samples	cDNA	Leave-one-out cv	–	–	82% accuracy	14973550 [20]
**EC05**	43	78 (32+46)	Overall survival in colorectal tumor samples	cDNA	Leave-one-out cv/Independent expression dataset	Prognosis not specified	95*	90% accuracy/Association	15908663 [21]
**HO09**	32	6 (3+3)	Cell lines were used to build a metastatic potential profile.	Affymetrix	Independent expression dataset (5 genes)	Overall survival	181*	Association	20077526 [22]
**JG08**	7	73 (42+31)	Recurrence in colorectal tumor samples	Affymetrix	Independent expression dataset/Independent RT-PCR	Recurrence	123 (105+18)/110 (86+24)	68% accuracy/82% accuracy	18556775 [23]
**JS09**	163	209 (86+123)	Duke’s A vs D colorectal tumor samples. Data set included 30 distant metastasis.	Affymetrix	2-fold cv	Recurrence and overall survival	99*	Association	19996206 [24]
**KL10**	36	100 (69+31)	Recurrence in rectum samples	Illumina Sentix Human-6 Expression Beadchip	5-fold cv/6-fold Montecarlo cv	–	–	71% accuracy/80% accuracy	20670856 [25]
**KN11**	634	215 (142+73)	Metastasis in colon tumor samples	cDNA	5-fold cv/Independent expression dataset	Metastasis	144 (85+59)	0.68 AUC/0.68 AUC	22067406 [26]
**LC09**	54	23 (9+14)	Only rectum samples used to build a chemoradiotherapy repondent signature	cDNA	No validation	–	–	–	19380020 [27]
**LN07G**	19	55 (29+26)	Recurrrence in colorectal tumor samples	Affymetrix	Independent expression dataset	Recurrrence	149 (102+47)	67% accuracy	17255271 [28]
**LN07NZ**	22	149 (102+47)	Recurrence in colorectal tumor samples	MWG 30 K Oligo Set	Independent expression dataset	Recurrrence	55 (29+26)	71% accuracy	17255271 [28]
**MT10**	12	48 (32+16)	Breast tumor samples were used to derive a instability profile.	cDNA	Three independent expression datasets	Metastasis	50 (25+25)/24 (14+10)	69–72% accuracy	21161944 [29]
**OC10**	7	1851*	Recurrence in colorectal tumor samples	RT-PCR	Independent RT-PCR [43]	Recurrence	1436 (1158+278)	Association	20679606 [30]
**PG10**	8	95 (58/37)	Recurrence in colorectal tumor samples	DASL Illumina Cancer Panel	No validation	–	–	–	20706727 [31]
**PL10**	7	74 (54+20)	Overall survival in colorectal tumor samples	RT-PCR	No validation	–	–	–	19901968 [32]
**SL10**	18	188 (137+51)	Metastasis in colorectal tumor samples	Agilent WG oligo hd	Four independent expression datasets	Recurrence	206*/100 (62+38)	Association	21098318 [33]
**SC09**	6	57*	Cancer specific survival in colorectal tumor samples	RT-PCR	2-fold cv/Independent RT-PCR	Cancer specific survival	83*	Association	19737943 [34]
**SM09**	34	55 (35+20)	Profile genes searched as indicated DNA replication.	Affymetrix	Independent expression dataset	Recurrence, cancer specific survival and overall survival	177 (103+71)/(122+55)/(104+73)	63–70% accuracy	19914252 [35]
**ST09**	113	159*	Profile derived as co-expressed with WIPF1.	Affymetrix	Independent expression dataset	Recurrence and overall survival	62 (47+13)/(50+12)	Association	19399471 [36]
**VL10**	163	232 (177+55)	Recurrence in colorectal tumor samples	Affymetrix	Leave-one-out cv Independent expression data set	Recurrence	60 (44+16)	Association	21119668 [37]
**WN10**	28	96 (59+37)	Prognostic profile derived from breast tumor samples	cDNA	Two independent expression datasets	Metastasis	50 (25+25)/24 (14+10)	94% accuracy/75% accuracy	20596637 [38]
**WG04**	23	38 (25+13)	Recurrence in colorectal tumor samples	Affymetrix	2-fold cv	–	–	78% accuracy	15051756 [39]
**WT09**	45	36 (23+13)	Recurrence in colorectal tumor samples	Affymetrix	9-fold Montecarlo cv	–	–	92% accuracy	19016304 [40]
**WT10**	10	160 (115+45)	Liver metastasis in colorectal tumor samples	Affymetrix	2-fold cv	–	–	86% accuracy	20570135 [41]
**YM06**	119	92 (32+60)	Metastasis in colorectal tumor samples. Data set included 34 liver metastasis	Colonochip	Independent expression dataset	Metastasis	28 (18+10)	93% accuracy	17143521 [42]

**Signature**: signature name; **Training dataset**: public training data set if used in this work; **Validation dataset**: public test data set if used in this work**; Signature size**: reported signature size in the original paper (genes or features):; **Training sample size (good + poor)**: sample size of training data set, separating good and poor prognosis when reported; **Training outcome**: outcome used to derive the signature; **Training platform**: platform used for the training data set; **Signature validation**: type of validation for signature if performed; **Independent validation outcome**: outcome used for independent validation if performed; V**alidation results:** for each validation performed, accuracy classification measures or association assessing if provided; **Reference**: PMID and reference for publishing paper. * Frequencies of subgroups were not available. **Abbreviations**: **TMA**: tissue microarray; **cv**: cross-validation; **ns**: not specified.

Because different platforms and feature identifiers were used in signatures and gene expression datasets, a translation into the official Gene Symbol was done in order to have a common annotation. This translation was performed using the Universal Protein Resource annotation database [46], the online repository of HUGO Gene Nomenclature Committee [47] and the chip annotation files from the Affymetrix web site [48]. Unavoidably, no match was found for some features in some datasets and they were lost for subsequent analysis (File S3).

### Statistical Analysis

Since follow-up time was not available for most of the datasets, a binary outcome was defined as a prognosis status (Table 2). Whenever possible, a minimum of three years of follow up was required for patients without tumor recurrence. Nevertheless, two datasets with no follow up information were included (GSE5206 and GSE10402) to increase the sample size. Stage IV individuals were included in the analysis as recurrence events as it was expected that the specific expression changes in poor prognostic samples remain unaltered in the primary tumor once the metastases has occurred. When data was available, subgroup analysis were performed according to stage and microsatellite instability status (MSS/MSI).

**Table 2:** Datasets description.

Dataset	Trained signatures	Validation signatures	Outcome	Minimum follow up	Number of samples (no event + event)	Clinical info*	Platform
**GSE5206**	ST09	SL10	Recurrence	Not available	100 (62+38)	Stage 0–4, MSI no info	Affymetrix
**GSE17537**	SM09, VL10^a^	–	Recurrence completed with specific survival	3 years	47 (27+20)	Stage 1–4, MSI (NA)	Affymetrix
**GSE17536**	VL10^a^	SM09	Recurrence completed with specific survival	3 years	141 (68+73)	Stage 1–4, MSI (NA)	Affymetrix
**GSE2630**	BD07	–	Recurrence	5 years	16 (10+6)	Stage 1–2, MSI no info	H 19K Oligo
**E-MEXP-1245**	LN07NZ	SL10	Recurrence	5 years	149 (102+47)	Stage 1–4 (NA), MSI no info	MWG H 30K
**GSE12945**	–	ST09	Recurrence	3 years	55 (42+13)	Stage 1–4, MSI no info	Affymetrix
**GSE10402**	–	SL10	Recurrence	Not available	73 (63+10)	Stage 1–3 (NA), MSI no info	Hs OperonV2
**GSE14333**	JS09^b^	VL10, JS09^b^	Recurrence	3 years	227 (116+111)	Stage 1–4, MSI no info	Affymetrix
**GSE13294**	–	–	Recurrence	3 years	146 (110+36)	Stage 1–4, MSI	Affymetrix
**GSE28722**	–	–	Metastasis	3 years	86 (51+35)	Stage 1–4, MSI no info	Rosetta 23K
**GSE18088**	–	–	Recurrence	5 years	53 (40+13)	Stage 2, MSI	Affymetrix

**Dataset:** GEO or Array Express dataset identifier; **Trained signatures:** signatures which used that dataset as training sample, if any; **Validation signatures:** signatures which used that dataset as independent validation sample; **Outcome:** type of relapse used for that dataset; **Minimum follow up:** minimum follow up required for that dataset, when this info was available; **Number of samples:** number of samples contained in that dataset, showing good and bad prognosis’ separately between brackets; **Clinical info**: samples ranges of stage and microsatellite status when this information was available; **Platform:** datasets’ hybridization platform. * NA: the authors do not provide clinical information about MSI and/or stage. No info: Although authors provide clinical information in the paper, samples are not labelled with this information in GEO or ArrayExpress. **a.** Stage II and III samples from data sets GSE17536 and GSE17537 were jointly used to derive signature VL10, but the later did not include enough events at these stage subgroups. **b.** Signature JS09 was built with Duke’s A and D and validated with Duke’s B and C samples.

Each signature’s ability to predict prognosis was independently tested in each dataset with a binary classification approach using the *Random Forest* ensemble classifier (RF) [49], [50]. Forests were grown with a high number of trees (5.000) in order to assure out-of-bag error convergence. The minimum size of terminal nodes was set to one. For signatures evaluation, accuracy measures were computed from a 10-fold cross-validation (10CV) process in which partitions were stratified based on outcome. Nested in this process, the number of candidate variables at each split was selected to minimize the out-of-bag error. As suggested in [51], sub-sampling was carried out without replacement and using the same number of observations in each prognosis group (0.632 times frequency of the smallest group). Due to lack of balance in outcome groups in some datasets, RF showed a trend to preferably classify into the most frequent group. To correct this artifact, the classification vote cutoff was modified according to the corresponding prognosis group frequencies. All these analyses were performed using the R package randomForest [52].

To confirm our results, a radial kernel Support Vector Machine (SVM) based classifier was also used [53], [54]. Due to unbalance, the same artifact described above was observed when applying the standard SVM classifier. So, we tried to correct it using an under-sampling strategy as follows [55]: i) select all samples from the less frequent group; ii) randomly select the same number of samples from the more frequent group; iii) repeat the process 25 times; iv) define the predicted labels using the outcome group frequencies as vote proportion cutoff for the classification rule. A 10CV process was carried out to compute accuracy measures with a nested 10CV for parameter tuning, both of them stratified by outcome groups. A wide range of values for cost and radial kernel parameters were evaluated during the tuning process (20 equidistant values from 0.001 to 1.000 in logarithm scale; 11 equidistant values ranging 0.05×*p* to 20×*p*, being *p* the number of features in each case). All these analysis were conducted with the R package *svmpath* [56].

The Matthews Correlation Coefficient (MCC) [57] was chosen as measure of classification accuracy [58]. This index combines test sensitivity and specificity. It ranges from −1 to 1 and its interpretation is similar to the Pearson’s correlation coefficient. In the context of a classification problem it is expected that MCC ranges from 0 (no prediction ability at all) to +1 (perfect prediction) with negative values near zero possibly occurring in random classifiers due to sample variability. MCC values lower than 0.3 can be considered as indicative of low predictive value as they correspond to less than 65% accuracy in balanced data. Sensitivity, specificity and overall accuracy rates were also computed for interpretation purposes.

The potential usefulness of the signatures on clinical practice was evaluated by means of the positive and negative likelihood ratios (LR+, LR-) and the predicted positive and negative post-test recurrence probabilities (PPTpr, NPTpr) in stage II and III samples separately [59].

To summarize signature’s global performance, each of the measures above was pooled across datasets to a unique index using weights proportional to each dataset sample size. In order to attenuate instability and bias in the cross-validation estimations, datasets with less than 10 samples per group and those used in the derivation of the profile in the original study were excluded from these computations [60].

Significance of MCC, accuracy, sensitivity, specificity, LR+, LR-, and differences of PPTpr and NPTpr were assessed using null distributions based on 100.000 permutations. Computations were done in the context of the theoretical framework for permutation tests [61] as implemented in the R package *coin* [62]. Intervals at 95% were built using the Bias Corrected and Accelerated bootstrap (BCa) method with 5.000 resamples stratified by prognosis group [63]. Empirical influence values were estimated by the usual jackknife method. These calculations were done using the R package *boot* [64]. In all cases, permutations and resampling were performed directly on the predicted values provided by the original models and no remodeling was done. Since this strategy doesn’t take into account the dependence in predictions implicitly imposed by the 10CV, it could potentially retain some bias towards refusing null hypothesis in the statistical tests when effects are small [65].

## Results

### Global Prognosis Performance of the Published Signatures

The literature search identified 29 papers reporting 31 signatures proposed as valid multi-gene tumor-outcome classification tool (Table 1 and File S4). Almost all signatures were based on microarray experiments with the exception of three signatures obtained from PCR experiments (*OC10, PL10, SC09*). The number of genes (signature size) ranged from 3 to 537.

Despite recommendations to provide raw data for microarray experiments, training datasets were publicly available only for five signatures: *ST09, SM09, BD07, LN07,* and *VL10*. Six additional gene expression datasets with information about recurrence were identified in GEO and ArrayExpress, for a total of 11 datasets available for analysis (Table 2).

**Figure 2:**
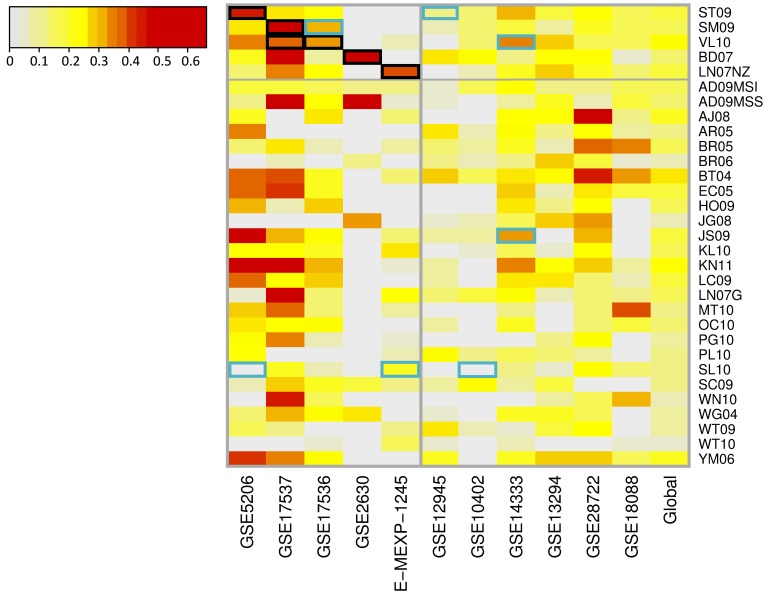
Heatmap showing Matthews Correlation Coefficient (MCC) values for each signature in each dataset as result of analyses with Random Forest. Rows correspond to signatures and columns to datasets. Last column shows a pooled MCC across datasets using sample size as weights. Black lines delimit the first five signatures for which training datasets were available (cells highlighted in black). Cells representing signatures and datasets used to validate them are highlighted in blue. Color scale represents the MCC values: the darker the color, the higher MCC (see the legend). Negative values were collapsed to zero.

For all combinations of signatures and datasets, the MCC and the corresponding p-value was computed. Figure 2 shows a color map of the MCC values (details provided in File S5 and Figure S1). As expected, the five signatures for which the training was available showed significant association and a reasonable predictive accuracy in their training datasets (black-highlighted cells at the top left quadrant in the plot in Figure 2). For all these signatures, MCC values were greater than 0.35 except for *VL10* and dataset GSE17536 (MCC = 0.32). Nevertheless, in the independent datasets the performance was heterogeneous and none of these five signatures could reproduce the degree of predictive ability shown in the training datasets. When the remaining 26 signatures (those without training set available) were evaluated in the 11 datasets, similar results were obtained: some signatures showed a significant association with patient outcome but discrimination accuracy was low or moderate.

A global MCC was computed for each signature to summarize their predictive ability across datasets (see Figure 2). Signatures *BT04* and *KN11* emerged as the most predictive, both with a MCC value of 0.25 (95% CI 0.19 to 0.31 and 0.19 to 0.30 respectively, p-values <10^5^). Although nearly all the signatures reached the 5% significance level in this pool estimate that combines 396 events in 1077 patients, only three signatures exceed a 0.20 global MCC. The maximum value obtained for the proportion of correctly classified cases was only 63% (*BT04,* sensitivity = 65% and specificity = 61%) and it ranged from 52 to 61% for the remaining profiles (Table 4, File S6).

**Table 3:** Clinical characteristics of datasets.

		GSE5206	GSE17537	GSE17536	GSE2630	E-MEXP-1245	GSE12945	GSE10402	GSE14333	GSE13294	GSE28722	GSE18088	Total
	**Good**	62 (62.0%)	27 (57.4%)	68 (48.2%)	10 (62.5%)	102 (68.5%)	42 (76.4%)	63 (86.3%)	116 (51.1%)	110 (75.3%)	51 (59.3%)	40 (75.5%)	691 (63.2%)
**Prognosis**	**Bad**	38 (38.0%)	20 (42.6%)	73 (51.8%)	6 (37.5%)	47 (31.5%)	13 (23.6%)	10 (13.7%)	111 (48.9%)	36 (24.7%)	35 (40.7%)	13 (24.5%)	402 (36.8%)
	**Total**	100 (100.0%)	47 (100.0%)	141 (100.0%)	16 (100.0%)	149 (100.0%)	55 (100.0%)	73 (100.0%)	227 (100.0%)	146 (100.0%)	86 (100.0%)	53 (100.0%)	1093 (100.0%)
	**Male**	46 (46.0%)	21 (44.7%)	79 (56.0%)	11 (68.8%)	70 (47.0%)	30 (54.5%)		133 (58.6%)	71 (50.4%)		26 (49.1%)	487 (52.4%)
**Gender**	**Female**	54 (54.0%)	26 (55.3%)	62 (44.0%)	5 (31.2%)	79 (53.0%)	25 (45.5%)		94 (41.4%)	70 (49.6%)		27 (50.9%)	442 (47.6%)
	**Total**	100 (100.0%)	47 (100.0%)	141 (100.0%)	16 (100.0%)	149 (100.0%)	55 (100.0%)		227 (100.0%)	141 (100.0%)		53 (100.0%)	929 (100.0%)
	**Miss**	0 (0.0%)	0 (0.0%)	0 (0.0%)	0 (0.0%)	0 (0.0%)	0 (0.0%)		0 (0.0%)	5 (3.4%)		0 (0.0%)	164 (15.0%)
**Age**	**Mean (Sd)**	64 (14.2)	61 (13.5)	65 (13.0)	64 (11.2)		64 (11.6)	67 (12.8)	65 (12.5)		63 (12.5)	65 (12.2)	65 (12.7)
	**I**	15 (15.5%)	4 (8.5%)	18 (12.8%)	6 (37.5%)		11 (20.0%)		31 (13.7%)	5 (3.4%)	13 (15.3%)	0 (0.0%)	103 (11.9%)
	**II**	29 (29.9%)	9 (19.1%)	38 (27.0%)	10 (62.5%)		23 (41.8%)		64 (28.2%)	123 (84.2%)	44 (51.8%)	53 (100.0%)	393 (45.3%)
**Stage**	**III**	33 (34.0%)	17 (36.2%)	46 (32.6%)	0 (0.0%)		16 (29.1%)		71 (31.3%)	10 (6.8%)	23 (27.1%)	0 (0.0%)	216 (24.9%)
	**IV**	20 (20.6%)	17 (36.2%)	39 (27.7%)	0 (0.0%)		5 (9.1%)		61 (26.9%)	8 (5.5%)	5 (5.9%)	0 (0.0%)	155 (17.9%)
	**Total**	97 (100.0%)	47 (100.0%)	141 (100.0%)	16 (100.0%)		55 (100.0%)		227 (100.0%)	146 (100.0%)	85 (100.0%)	53 (100.0%)	867 (100.0%)
	**Miss**	3 (3.0%)	0 (0.0%)	0 (0.0%)	0 (0.0%)		0 (0.0%)		0 (0.0%)	0 (0.0%)	1 (1.2%)	0 (0.0%)	226 (20.7%)
	**Colon**	75 (75.0%)			16 (100.0%)	149 (100.0%)	26 (47.3%)	73 (100.0%)	193 (85.4%)	121 (82.9%)	72 (83.7%)	53 (100.0%)	778 (86.1%)
**Site**	**Rectum**	25 (25.0%)			0 (0.0%)	0 (0.0%)	29 (52.7%)	0 (0.0%)	33 (14.6%)	25 (17.1%)	14 (16.3%)	0 (0.0%)	126 (13.9%)
	**Total**	100 (100.0%)			16 (100.0%)	149 (100.0%)	55 (100.0%)	73 (100.0%)	226 (100.0%)	146 (100.0%)	86 (100.0%)	53 (100.0%)	904 (100.0%)
	**Miss**	0 (0.0%)			0 (0.0%)	0 (0.0%)	0 (0.0%)	0 (0.0%)	1 (0.4%)	0 (0.0%)	0 (0.0%)	0 (0.0%)	189 (17.3%)
	**MSS**									73 (50.0%)		34 (64.2%)	107 (53.8%)
**Microsatellite**	**MSI**									73 (50.0%)		19 (35.8%)	92 (46.2%)
**stability**	**Total**									146 (100.0%)		53 (100.0%)	199 (100.0%)
	**Miss**									0 (0.0%)		0 (0.0%)	894 (81.8%)
	**Well**	8 (8.3%)	1 (3.1%)	12 (8.5%)			0 (0.0%)					2 (3.8%)	23 (7.1%)
	**Moderately**	78 (81.2%)	24 (75.0%)	105 (74.5%)			28 (50.9%)					35 (66.0%)	242 (75.2%)
**Grade**	**Poorly**	10 (10.4%)	7 (21.9%)	24 (17.0%)			27 (49.1%)					16 (30.2%)	57 (17.7%)
	**Total**	96 (100.0%)	32 (100.0%)	141 (100.0%)			55 (100.0%)					53 (100.0%)	322 (100.0%)
	**Miss**	4 (4.0%)	15 (31.9%)	0 (0.0%)			0 (0.0%)					0 (0.0%)	771 (70.5%)

To assess influence of the statistical methodology in the results, a re-analyses was performed using an alternative method (SVM). Although some variations in the signatures ranking of performance were observed, similar results were obtained in terms of pooled MCCs (Figure S2 and File S7).

### Subgroup Analysis: Prognosis Performance of Published Signatures Stratified by Stage or MSI Status

In order to assess the signatures’ performance in specific subgroups of tumors, a stratified analysis was done according to stage (stage II/stage III) and microsatellite instability status (MSS/MSI), when this information was available (see Table 3). Datasets contributing with less than 10 events were excluded.

**Table 4:** Global performance of top 10 signatures for all, stage II and stage III samples.

ALL SAMPLES			STAGE II					STAGE III				
Signature	MCC	Accuracy (Sensitivity, Specificity)	Signature	MCC	Accuracy (Senitivity, Specificity)	Positive Post-Test Probability	Negative Post-Test Probability	Signature	MCC	Accuracy (Senitivity, Specificity)	Positive Post-Test Probability	Negative Post-Test Probability
**BT04**	**0.254**	63% (65%, 61%)	**YM06**	**0.209**	58% (69%, 55%)	28%	12%	**AJ08**	**0.415**	71% (69%, 72%)	56%	18%
**KN11**	**0.247**	61% (65%, 60%)	**BT04**	**0.202**	59% (66%, 57%)	28%	13%	**VL10**	**0.400**	70% (68%, 72%)	55%	19%
**VL10**	**0.212**	60% (63%, 59%)	**EC05**	**0.199**	58% (68%, 56%)	28%	13%	**KN11**	**0.394**	69% (70%, 67%)	53%	18%
**YM06**	**0.192**	59% (61%, 58%)	**KN11**	**0.187**	57% (68%, 54%)	27%	13%	**JS09**	**0.385**	69% (70%, 67%)	52%	19%
**AJ08**	**0.190**	59% (63%, 57%)	**VL10**	**0.181**	58% (66%, 55%)	27%	13%	**LC09**	**0.324**	66% (69%, 63%)	49%	20%
**ST09**	**0.178**	58% (61%, 56%)	**LN07NZ**	**0.179**	59% (64%, 58%)	27%	14%	**BT04**	**0.322**	66% (65%, 66%)	50%	21%
**LN07NZ**	**0.176**	59% (61%, 57%)	**BR05**	**0.172**	59% (62%, 58%)	27%	14%	**SM09**	**0.312**	65% (68%, 63%)	49%	21%
**LC09**	**0.171**	58% (60%, 57%)	**MT10**	**0.158**	57% (65%, 54%)	26%	14%	**YM06**	**0.307**	64% (67%, 62%)	47%	22%
**EC05**	**0.167**	58% (60%, 56%)	**SM09**	**0.153**	56% (66%, 52%)	26%	14%	**OC10**	**0.298**	64% (64%, 64%)	48%	22%
**JS09**	**0.165**	57% (62%, 55%)	**LC09**	**0.151**	53% (69%, 49%)	25%	14%	**KL10**	**0.292**	64% (68%, 60%)	47%	22%

**Abbreviations**: **MCC**: Matthews Correlation Coefficient.

Similar to the analysis including all samples, the performance of the signatures was heterogeneous when stage II and III tumor samples were analyzed separately (Figures S3 and S4). In the pooled MCC, 17 signatures in stage II and 22 signatures in stage III showed a significant association with prognosis (p-value <0.05). Six signatures ranked in the top ten in both sub-analyses. The MCC values obtained in stage II were much lower than those in stage III. In stage II, the best global MCC were achieved by *YM06* (MCC = 0.21; 95%CI 0.11 to 0.31) and *BT04* (MCC = 0.20; 95%CI 0.10 to 0.31). In stage III, the two best signatures were *AJ08* (MCC = 0.42; 95%CI 0.28 to 0.55) and *VL10* (MCC = 0.40; 95%CI 0.23 to 0.55). Table 4, Files S5, S6 and S7; and Figures S3, S4, S5, S6, S7 and S8 contain more details.

MSI status information was only available for two datasets (GSE13294 and GSE18088). In the analysis of MSS samples, those MCC values that reached significance were moderate (0.19 to 0.38) and only three signatures showed association in both datasets (p-value <0.1). Regarding the MSI subset, only signature *HO09* provided a reasonably classification accuracy (MCC = 0.30) (File S5).

### Potential Clinical Value of Signatures in Stage II and III Tumors

Despite the low discrimination ability (shown by their pooled MCC), the signatures could still have usefulness in clinical practice. Briefly, a useful clinical test typically shows large LR+ and low LR- which translate into more discriminant post-test event probabilities: high PPTpr and low NPTpr compared to the *a priori* expected event proportion. So, even tests with low discrimination ability according to pure statistic criteria could still be useful in clinical practice if PPTpr and NPTpr are significantly far enough from the probability expected in population when no test is performed. To explore this issue, positive and negative post-test probabilities of recurrence were calculated for stages II and III. The prior recurrence risk in patients with CRC was assumed to be 20% in stage II and 34% in stage III [4], [66].

**Figure 3:**
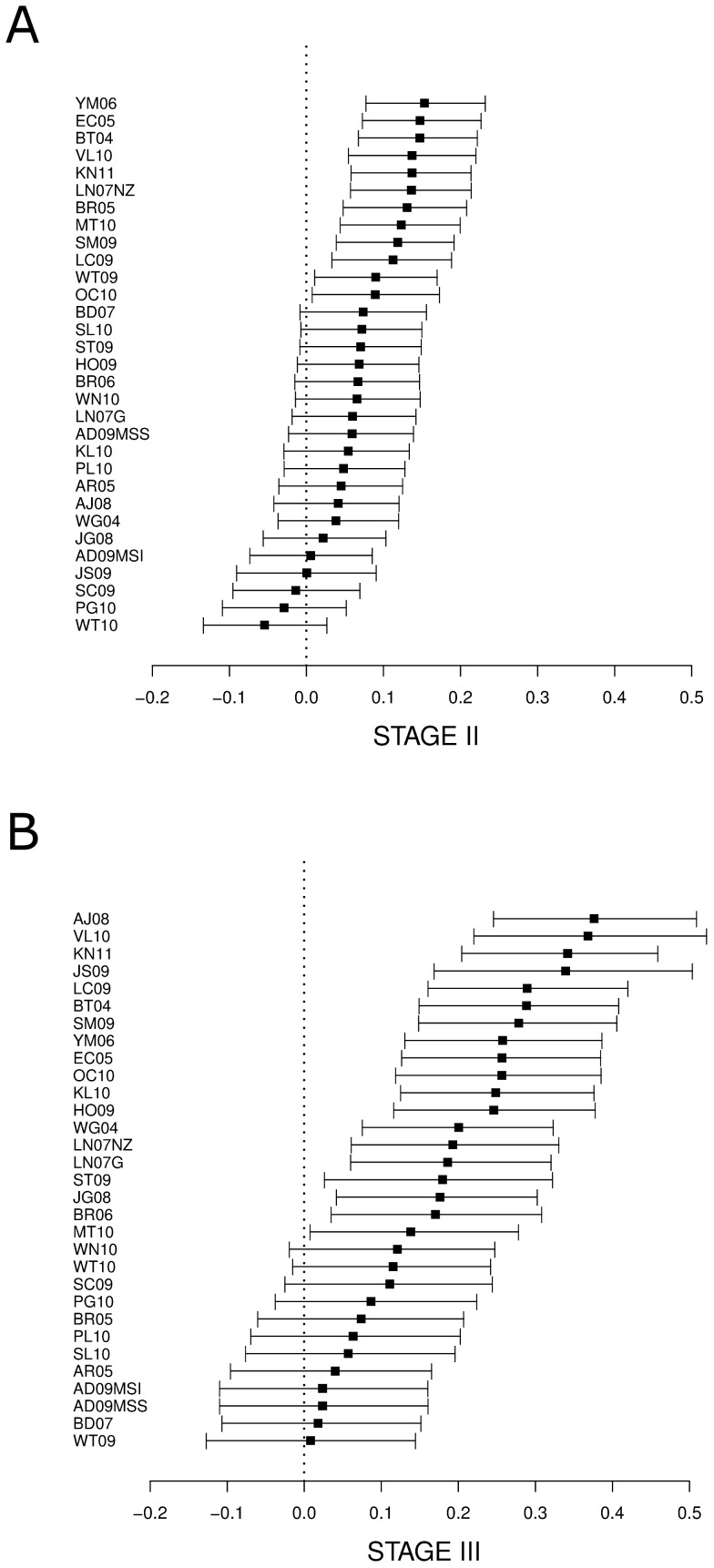
Differences between positive and negative post-test probabilities of recurrence and their 95% confidence interval for stage II (A) and stage III (B). Prevalence probability of recurrence for stage II and III were assumed to be 20 and 34% respectively. Signatures are listed in decreasing order of post-tests probability differences.

For the best signature in stage II (*YM06*), the post-test recurrence probability for the high-risk group increased to 28%, and for the low-risk group the prediction was 12% probability of recurrence (16% absolute difference, Figure 3A). The best profile in stage III (*AJ08*) increased to 56% the post-test probability of recurrence for the high-risk group, while the post-test probability was 18% for the low-risk group (38% absolute difference, Figure 3B). Detailed results for all signatures are shown in Files S6 and S7.

## Discussion

The identification of molecular prognostic tools to facilitate treatment decisions is an important step for individualized patient therapy [10]. Here we report an exhaustive analysis of published multi-gene prognostic classifiers in colorectal cancer, analyzing their external validity in a large number of independent datasets that total more than 1.000 patients. The present work is focused in two objectives which are addressed by the two main parts of the analysis: to evaluate the global performance of the signatures from a statistical point of view, in which all stages were included, and to assess their potential clinical usefulness, restricted to stage II and III CRC patients, by means of appropriate accuracy measures (post-test probabilities).

A meta-analysis of gene expression profiles in stage II CRC has been previously reported by Lu *et al.* [67]. In that study, promising results were reported regarding predictive accuracy, but the analysis was confined to the same datasets and predictions used in the original studies. To our knowledge, our study is the first meta-analysis in which prediction accuracy of many signatures is measured in a large number of independent CRC samples to assess external validity and their subsequently potential usefulness in clinics.

In terms of global performance, our results indicate that in their training dataset, most signatures showed a significant association with prognosis and could reasonably predict the outcome. However, none of the signatures performed satisfactorily when the prediction ability was assessed in independent datasets. The best pooled MCC was 0.25 (*BT04*), which should be considered a low classification value. As a reference, stage provides an MCC of 0.23 (data not shown).

Next, we focused in specific performance of signatures in stage II and III patients, who could benefit more of an accurate prognosis prediction since adjuvant chemotherapy could be tailored to their predicted recurrence risk. Although association with outcome was observed for 17 signatures in stage II, their predictive ability can only be considered poor from a statistical point of view. Otherwise, MCC values in stage III were observed to be near double those in stage II. Nevertheless, only eight signatures achieved a 0.30 pooled MCC value, considered as indicative of moderate predictive value.

Although poor results were observed in terms of classification accuracy, almost all profiles (30) showed a significant association with prognosis when tested in independent datasets (p-value <0.05). Notice however that significant association only means that a signature prediction is not completely random (MCC = 0). Association is not sufficient to be useful since, with enough sample size, small effects can be significant. Better indicators of potential usefulness than significance are magnitude of the sensitivity and specificity or derived measures like the MCC or likelihood ratios, which measure the ability to correctly classify patients by their outcome.

Despite these disappointing results according to pure statistic criteria of discrimination ability, signatures might still be useful in clinical practice if they provide additional risk stratification within known sub-populations defined by relevant clinical variables. The positive and negative post-test probabilities of recurrence were calculated stratified by stage to identify the degree of prognosis discrimination beyond stage. The results for the best signature in stage II samples (*YM06*) moderately modified the 20% a priory recurrence probability to a 28% and 12% for the positive and negative post-test result, respectively. This discriminating ability is not completely satisfactory given the large false negative and positive rates that it would induce, but might contribute to the identification of stage II patients at high risk for recurrence leading to a better indication of adjuvant chemotherapy [6]. The best signature for stage III patients (*AJ08*) resulted in a larger discrimination of risk groups, with a difference between positive and negative post-test probabilities of 38%. However, the low risk group still showed a relatively large recurrence probability of 18%, too high to recommend avoiding adjuvant chemotherapy as it is indicated nowadays.

Potential explanations for these modest results must be considered. From a statistical point of view, technical problems such as low sample size, the number of genes included in the classifier, translation between platforms or cohort heterogeneity, among others, have been reported as potential explanations for the lack of clinical translation of genomic classifiers (see references [68], [69], [70]). In our case and for some signatures, only association with prognosis was reported in the original work, thus the authors implicitly recognized poor classification ability. In those profiles that were reported to be highly discriminative, the reason could be a poor control of over-fitting in the training methodology, since external validation was performed only in three studies and the test samples sizes were small (*JG08*, *WN10* and *YM06*, see Table 1). The need to map probes to genes for signatures that had used different platforms may also have affected the results, since it is known that even multiple probes of the same gene in the same platform may show important variability. We could not detect, however, that platform had a relevant effect in the MCC estimates.

Low availability of information and heterogeneity in clinical data is inherent to the use of public datasets and this is a major impediment for repeatability and integration of published microarray studies [71]. Datasets differ in patient characteristics, inclusion criteria and outcome definitions. A precise and homogeneous definition of the outcome across datasets would be desirable in order to obtain an accurate estimation of the signatures’ prognosis ability. Nevertheless, heterogeneity of datasets allows for a more pragmatic analysis and the estimates should reflect the expected results when profiles were used in real practice, since hospital settings are also heterogeneous. Since different outcomes are supposed to be highly correlated [72], we decided to prioritize a minimum sample size availability to get more precise estimates and avoid uncertainty introduced by datasets with less than 10 events [60]. The requirement of a three years minimum follow up also allowed maximizing sample size and was supported by the literature: it has been described that most of these relapses occur within 3 years after surgery and it is recommended to be used as endpoint in adjuvant clinical trials [73]. Therapy regimen followed by treated patients was not considered, as this information was not available for most of the analyzed datasets.

Patients diagnosed at stage IV were included and considered recurrent events to assess global performance. This implicitly assumes that the molecular changes playing a driver role to disease relapse remain unchanged in primary tumor after recurrence has occurred. Though this underlying hypothesis could be questionable, it was shared by many of the original studies analyzed that included stage IV subjects in their training and test datasets (*AJ08*, *BT04*, *EC05*, *HO09*, *JS09*, *LN07G*, *LN07NZ*, *PL10*, *SC09*, *SL10*, *SM09*, *ST09*, *VL10* and *YM06*).

From a biological perspective, this moderate prognosis ability could be explained by heterogeneity in tumor cell populations that might dilute the prognosis molecular signal. It is well known that CRC tumors are composed not only by tumor epithelial cells but also by cancer-associated stromal fibroblasts (CAFs), endothelial cells or inflammatory cells, among others [74]. Moreover, those cancer cells at the invasive front are different from those in the main tumor mass [75]. The problem of the tumor bulk heterogeneity can be overcome by isolating specific cells populations by laser microdissection technology [76]. In this regard, one out of the eleven sample sets used in this study (GSE12945) used this technique to specifically hybridize RNA from tumor cells. Surgical specimens from other sample sets were reviewed by a pathologist to assess a minimum tumor content of 80% (GSE5206, GSE18088, E-MEXP-1245). However, we did not observe significant differences in signatures performance regarding the tumor-cell enrichment method used.

The gene lists included in this study had little overlap: out of 1.530 genes reported in the 31 profiles, only two were shared by four signatures; 10 were shared by three signatures and 102 were present in two profiles. This result was not unexpected, since it has previously been reported [77], [78]. The lack of gene overlap is generally interpreted as if each signature is random sampling of a small subset of genes from a larger signature that represent the involved pathways [79], [80].

Colon and rectum tumors have been included indiscriminately in this work since in a previous report we showed that no significant differences exist between colon and rectum tumors at transcriptomic level [81]. However, this decision might explain some of the poor performance of the signatures, since it is known that surgery quality is an important prognostic factor in rectal cancer and less important in colon [82]. In the data used in this work, no significant association was found between prognosis and tumor location (data not shown).

The choice of the statistical tools for analyses was an important matter. The intention of this analysis was to test the performance of published prognostic signatures in independent datasets rather than trying to reproduce them using the original methodology. In this context, *Random Forest* arises as an efficient method that performs very well compared with other competitors [49], [83]. As expected, the signatures tested in their training dataset showed the highest accuracy. Moreover, we succeeded in reproducing the validation results of three out of the five signatures for which data was available (*SM09*, *VL10* and *JS09*). However, association with prognosis was not observed for profile *ST09* in dataset GSE12945, and it was only observed in one out of the three independent validation datasets that are included in this work for profile *SL10*, although good performance was originally reported (see Table 1, Figure 2 and File S5). A reason could be that the methodology we used does not capture well the prognosis value of some signatures, which might have been developed with more elaborated algorithms to define the risk prediction in the original study. Because this was a recognized limitation of this work, analyses were redone using an alternative methodology (SVM), which provided similar results (Figures S2, S5 and S6, File S7). In *ST09* profile, a semi-supervised approach was used while in *SL10* a nearest-centroid approach was applied which was not properly described in the paper. *SL10* was developed in an Agilent platform and the mapping of the probes to different validation platforms used in the datasets might be an addition source of divergence.

Although some works reported that simpler methods for supervised learning in the context of high-dimensional molecular data could perform equally than those used in this paper which are more elaborated [84], we chose RF and SVM because they are reported to be robust to over-fitting and the presence of noise, and they capable to learn complex classification functions. These properties are especially desirable in our study as we try to capture the hypothetical prediction ability of signatures created with very heterogeneous methodologies [49], [50], [52], [53]. So, our choice of these methodologies reflects our efforts in finding the prognosis information reported in the original works, though we may have failed in some complex signatures.

It is worth noting that two of the analyzed signatures correspond to current available commercial test for CRC prognosis. Oncotype DX was derived from *OC10* profile [85] and, interestingly, the reported risk estimations for strata in stage II in their validation study [43] were similar to those obtained in our work (PPTpr = 25% and 22%, NPTpr = 12% and 16% respectively). The algorithm for risk estimation with Oncotype DX implies the use of additional clinical information as tumor extent and mismatch repair status, which substantially improves its risk stratification. To our knowledge, no validation results for stage III patients have been published yet. Coloprint test was derived from *SL10*, which showed a low performance in our analysis, possibly for the reasons discussed above.

The characteristics of the available test datasets could be other reason of poor performance. Intriguingly, in some datasets (e.g. GSE17537) the performance of signatures was better than for others. This effect was not due to sample size neither tumor cell enrichment: Datasets with the largest number of events (GSE14333 and GSE13294) were not well classified by any of the tested signatures, and datasets with high tumor cell content showed uneven performance (e.g. GSE12945, GSE5206).

### Conclusions

Although most of the published signatures of prognosis in CRC tested in this analysis have shown significant statistical association with prognosis, their ability to accurately classify independent samples into high-risk and low-risk groups is limited. Thus, even when prognosis differences exist in expression data, higher accuracy is needed to consider a signature useful for the clinical practice. Well-designed studies, with large sample size, and preferably prospective are needed to accurately identify those patients at risk of recurrence, especially among patients with stage II CRC tumors.

## Supporting Information

Figure S1 Boxplots showing signatures’ MCC values in each dataset and pooled MCC. Dataset GSE2630 was excluded from pooled analysis due to low sample size.
(PDF)

Figure S2 Heatmap showing Matthews Correlation Coefficient values (MCC) for each signature in each dataset as result of analyses with Support Vector Machine. Rows correspond to signatures and columns to datasets. Last column shows a pooled MCC across datasets using sample size as weights. Black lines delimit the first five signatures for which training datasets were available (cells highlighted in black). Cells representing signatures and datasets used to validate them are highlighted in blue. Color scale represents the MCC values: the darker the color, the higher MCC (see the legend). Negative values were collapsed to zero.
(PDF)

Figure S3 Heatmap showing Matthews Correlation Coefficient (MCC) in stage II tumors as result of analyses with Random Forest. Empty columns are placed in case of no available data and datasets with less than 10 events, which were excluded from analyses.
(PDF)

Figure S4 Heatmap showing Matthews Correlation Coefficient (MCC) in stage III tumors as result of analyses with Random Forest. Empty columns are placed in case of no available data and datasets with less than 10 events, which were excluded from analyses.
(PDF)

Figure S5 Heatmap showing Matthews Correlation Coefficient (MCC) in stage II tumors as result of analyses with Support Vector Machine. Empty columns are placed in case of no available data and datasets with less than 10 events, which were excluded from analyses.
(PDF)

Figure S6 Heatmap showing Matthews Correlation Coefficient (MCC) in stage III tumors as result of analyses with Support Vector Machine. Empty columns are placed in case of no available data and datasets with less than 10 events, which were excluded from analyses.
(PDF)

Figure S7 Example of outcome association in stage II samples using disease free survival information: Kaplan-Meier estimates for risk groups predicted by signature Y*M06* in GSE13294 dataset (Random Forest results).
(PDF)

Figure S8 Example of outcome association in stage III samples using disease free survival information: Kaplan-Meier estimates for risk groups predicted by signature *AJ08* in GSE14333 dataset (Random Forest results).
(PDF)

Table S1 Excluded papers by eligibility criteria in the literature review.
(PDF)

File S1 Details on the prognosis signatures studies systematic review containing inclusion and exclusion criteria at each step.
(PDF)

File S2 The 27 PRISMA checklist items corresponding to the prognosis signatures studies systematic review.
(PDF)

File S3 Signatures translation results. Translation results for each signature to the platforms of public datasets used in this work: Affymetryx, Hs-OperonV2-vB2.2, Human 19 K Oligo array, MWG 30 K Oligo set and Rosetta custom human 23 K array. Translation was performed via Gene Symbol when necessary, using the Universal Protein Resource annotation database, the online repository of HUGO Gene Nomenclature Committee and the chip annotation files from the Affymetrix official web site. **Signature:** signature name; **Platform:** platform used to derive the signature; **Reported size:** size of signature reported in the original paper (genes or features); **Extracted size:** size of signature after extraction from the original paper (genes or features); **Gene Symbols**: size of signature in terms of official Gene Symbol when translation was possible; **Not found:** number of signature features not found in the platform; **% not found:** percentage of signature features not found in the platform (respect to extracted size); **Platform features**: signature size in the platform after translation. Signatures are listed in decreasing order of *% not found*.
(XLS)

File S4 Signatures official Gene Symbols and overlapping. ***Signatures Gene Symbol*** **:** signatures in terms of Gene Symbol. For each signature, official Gene Symbols to which some of their original feature was translated are listed. Translation was performed using the Universal Protein Resource annotation database, the online repository of HUGO Gene Nomenclature Committee and the chip annotation files from the Affymetrix official web site. ***Signatures overlap***: official Gene Symbols shared by four, three and two of the signatures used in this work. List of no shared Gene Symbols (*Genes in 1 signatures*) is also shown.
(XLS)

File S5 Random Forest classification results. Random Forest classification results for each signature and dataset are shown for all, stage II, stage III, Microsatellite Stable and Microsatellite instable samples analyses. **MCC**: Matthews Correlation Coefficient (MCC) and 95% confidence interval; **p-value**: permutation p-value associated with MCC; **Acc:** accuracy rate; **Sens**: sensitivity; **Spec**: specificity; **No events/events**: number of samples with good and bad prognosis respectively. Last column shows the same values for the pooled analyses across datasets using sample size as weights.
(XLS)

File S6 Global performance of signatures for all, stage II and stage III samples using Random Forest classifier. For each signature, sample size used in the analysis (separately for good and bad prognosis between brackets), pooled Matthews Correlation Coefficients (MCC) with 95% confidence intervals, accuracy rates, sensitivities and specificities are shown. For stage II and stage III analyses, also positive and negative likelihood ratios, negative and positive post-test probabilities, differences between post-test probabilities and 95% confidence interval are reported. Signatures are listed in decreasing order of MCC. Those with significant MCC at 5% level are highlighted in bold letters.
(XLS)

File S7 Support Vector Machine classification results. **Sheets** ***All samples*** **,** ***Stage 2*** **and** ***Stage 3:*** Support Vector Machine classification results for each signature and dataset are shown for all, stage II and, stage III analyses sheets respectively. **MCC**: Matthews Correlation Coefficient (MCC) and 95% confidence interval; **p-value**: permutation p-value associated with MCC; **Acc:** accuracy rate; **Sens**: sensitivity; **Spec**: specificity; **No events/events**: number of samples with good and bad prognosis respectively in that dataset. **Sheets** ***All samples global*** **,** ***Stage 2 global*** **and** ***Stage 3 global*** **:** Global performance results of signatures for all, stage II and stage III samples using Support Vector Machine are extended. Signatures are listed in decreasing order of MCC. Those with significant MCC at 5% level are highlighted in bold letters.
(XLS)
