# Supplementary figures and images for: Clinical Value of Prognosis Gene Expression Signatures in Colorectal Cancer: A Systematic Review
Source: PLoS One. 2012 Nov 7;7(11):e48877. doi: 10.1371/journal.pone.0048877 (PMC3492249; doi:10.1371/journal.pone.0048877)

**MCC values for each dataset**  
**All samples**

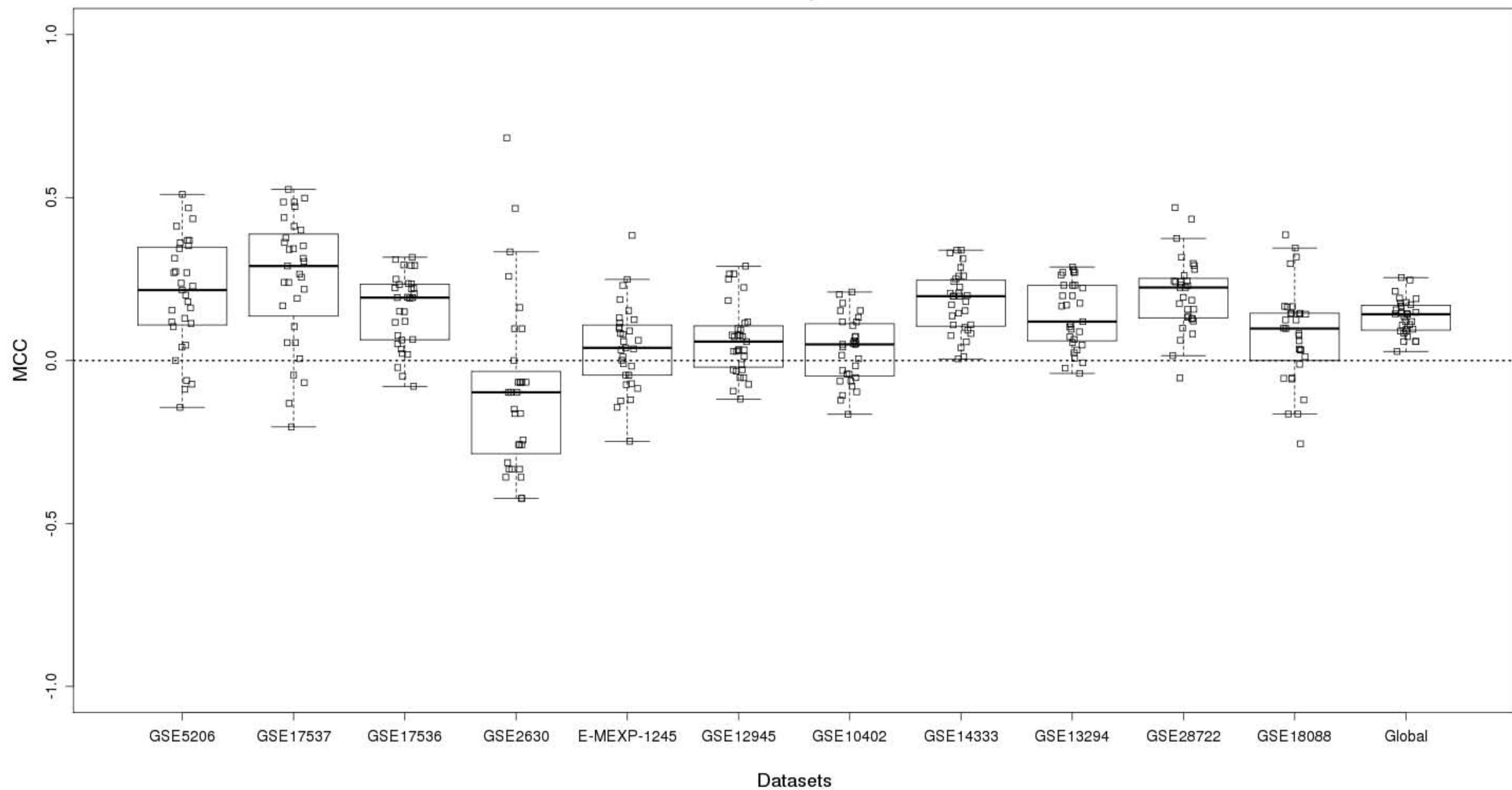

Supplement: Figure S1 — Boxplots showing signatures’ MCC values in each dataset and pooled MCC. Dataset GSE2630 was excluded from pooled analysis due to low sample size. (PDF) [file pone.0048877.s001.pdf]

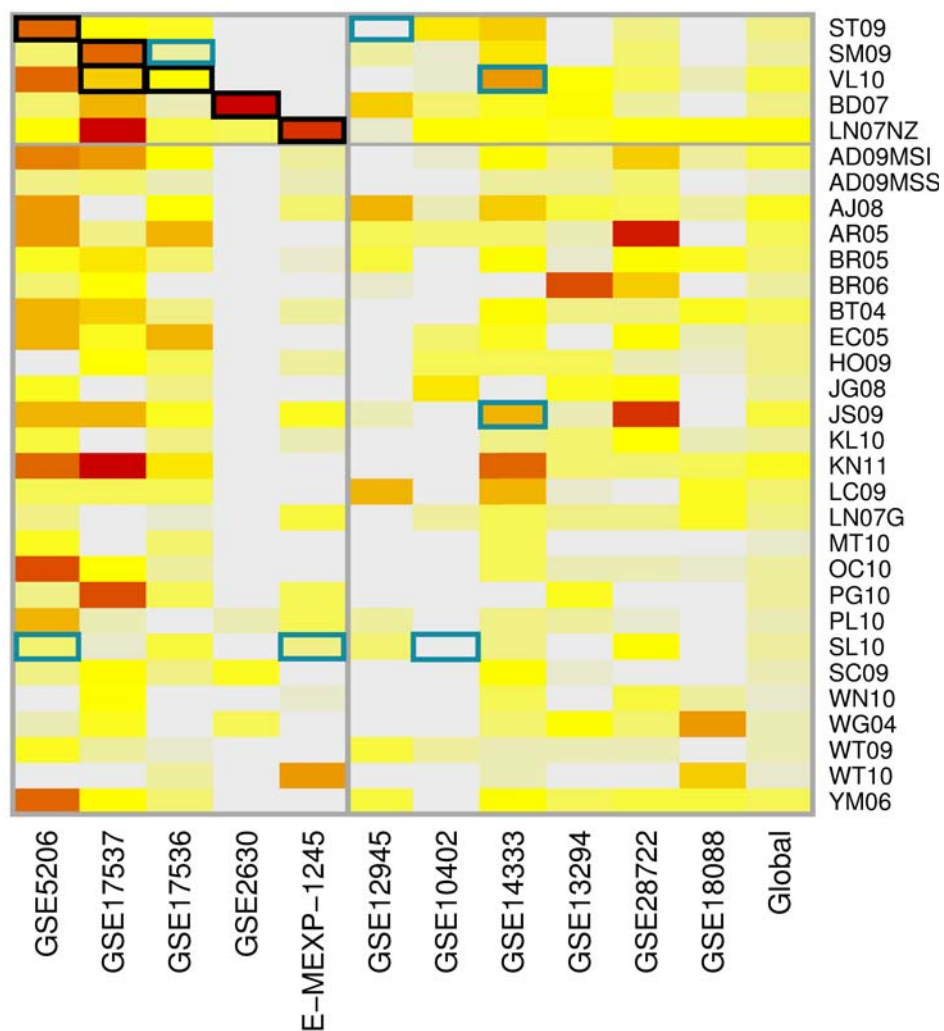

Supplement: Figure S2 — Heatmap showing Matthews Correlation Coefficient values (MCC) for each signature in each dataset as result of analyses with Support Vector Machine. Rows correspond to signatures and columns to datasets. Last column shows a pooled MCC across datasets using sample size as weights. Black lines delimit the first five signatures for which training datasets were available (cells highlighted in black). Cells representing signatures and datasets used to validate them are highlighted in blue. Color scale represents the MCC values: the darker the color, the higher MCC (see the legend). Negative values were collapsed to zero. (PDF) [file pone.0048877.s002.pdf]

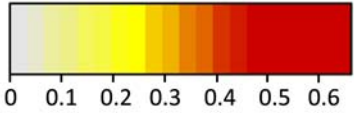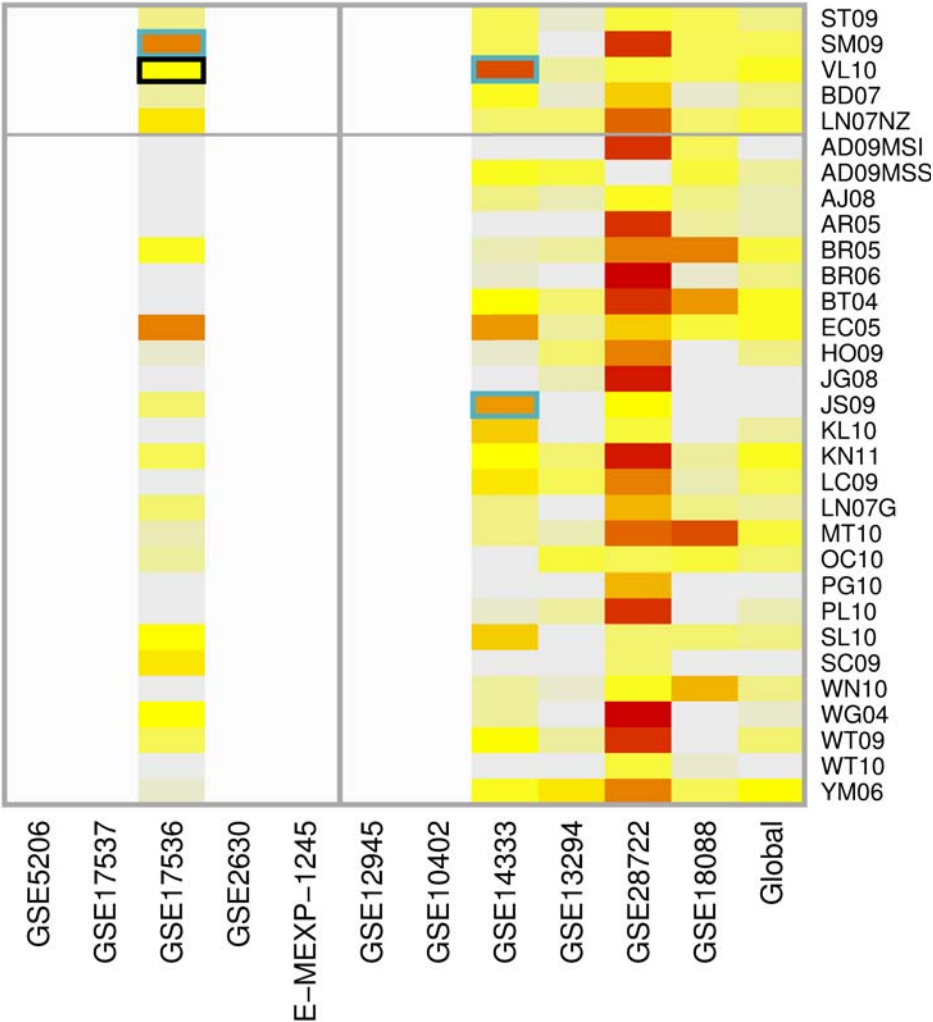

Supplement: Figure S3 — Heatmap showing Matthews Correlation Coefficient (MCC) in stage II tumors as result of analyses with Random Forest. Empty columns are placed in case of no available data and datasets with less than 10 events, which were excluded from analyses. (PDF) [file pone.0048877.s003.pdf]

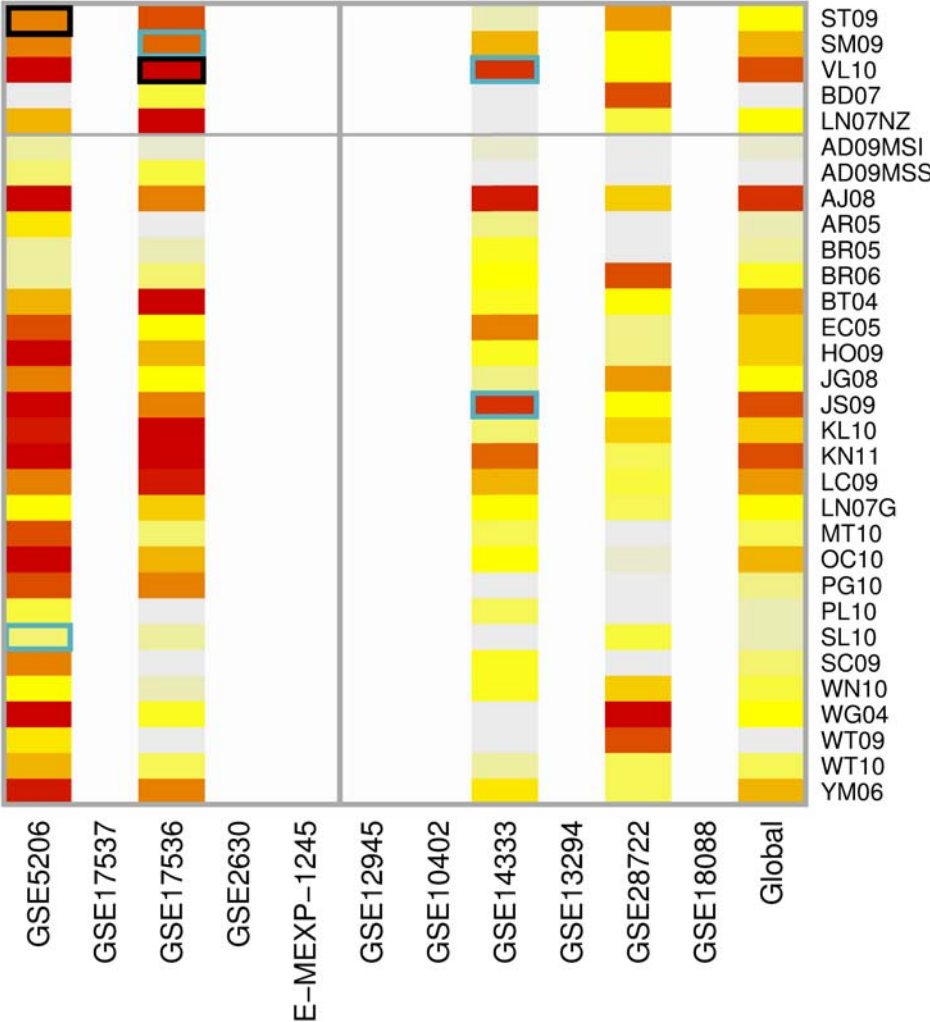

Supplement: Figure S4 — Heatmap showing Matthews Correlation Coefficient (MCC) in stage III tumors as result of analyses with Random Forest. Empty columns are placed in case of no available data and datasets with less than 10 events, which were excluded from analyses. (PDF) [file pone.0048877.s004.pdf]

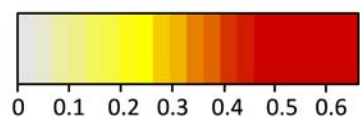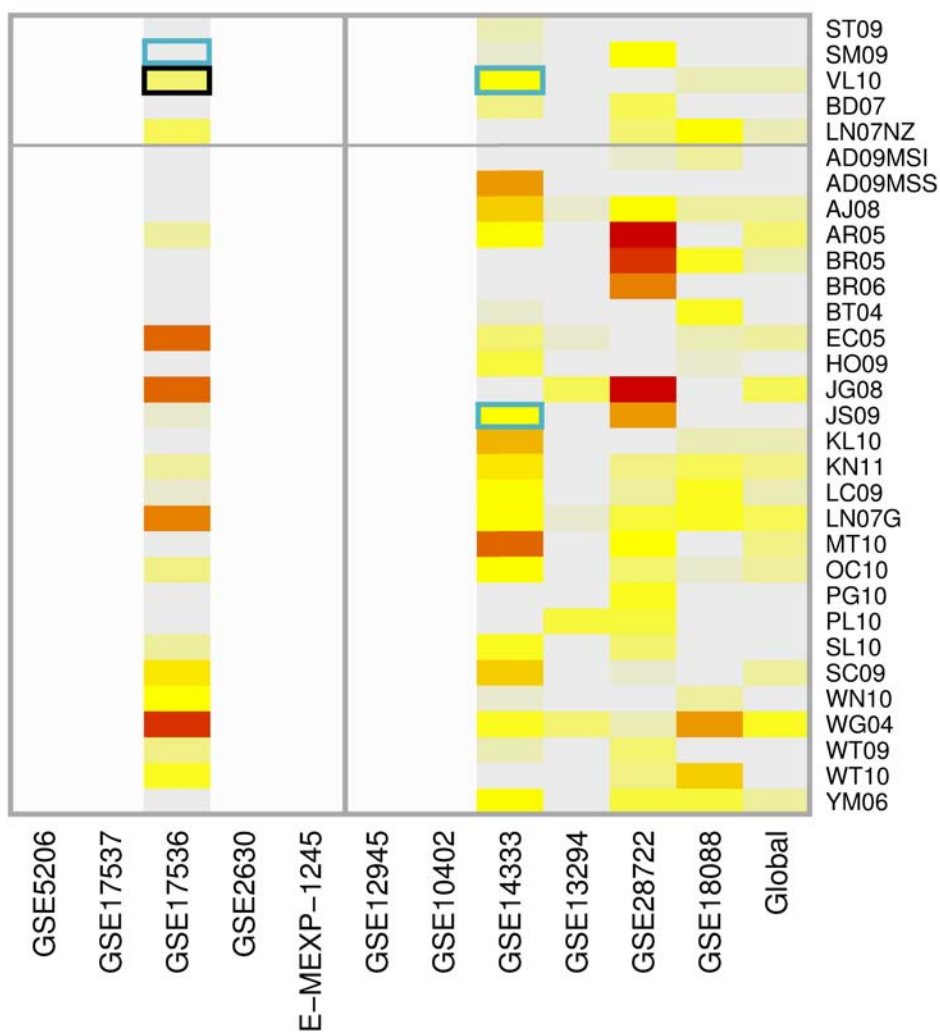

Supplement: Figure S5 — Heatmap showing Matthews Correlation Coefficient (MCC) in stage II tumors as result of analyses with Support Vector Machine. Empty columns are placed in case of no available data and datasets with less than 10 events, which were excluded from analyses. (PDF) [file pone.0048877.s005.pdf]

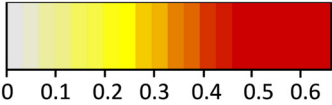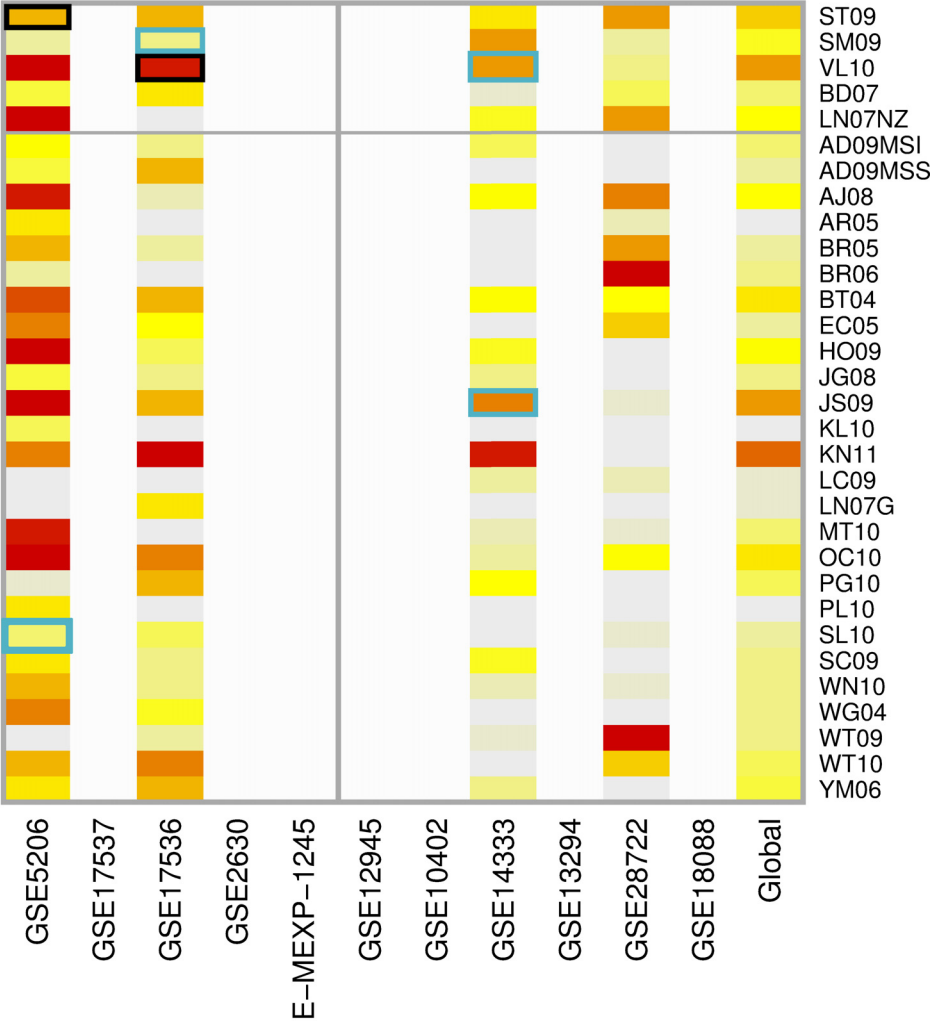

Supplement: Figure S6 — Heatmap showing Matthews Correlation Coefficient (MCC) in stage III tumors as result of analyses with Support Vector Machine. Empty columns are placed in case of no available data and datasets with less than 10 events, which were excluded from analyses. (PDF) [file pone.0048877.s006.pdf]

# Disease free survival for signature YM06 in dataset GSE13294

## Stage II samples

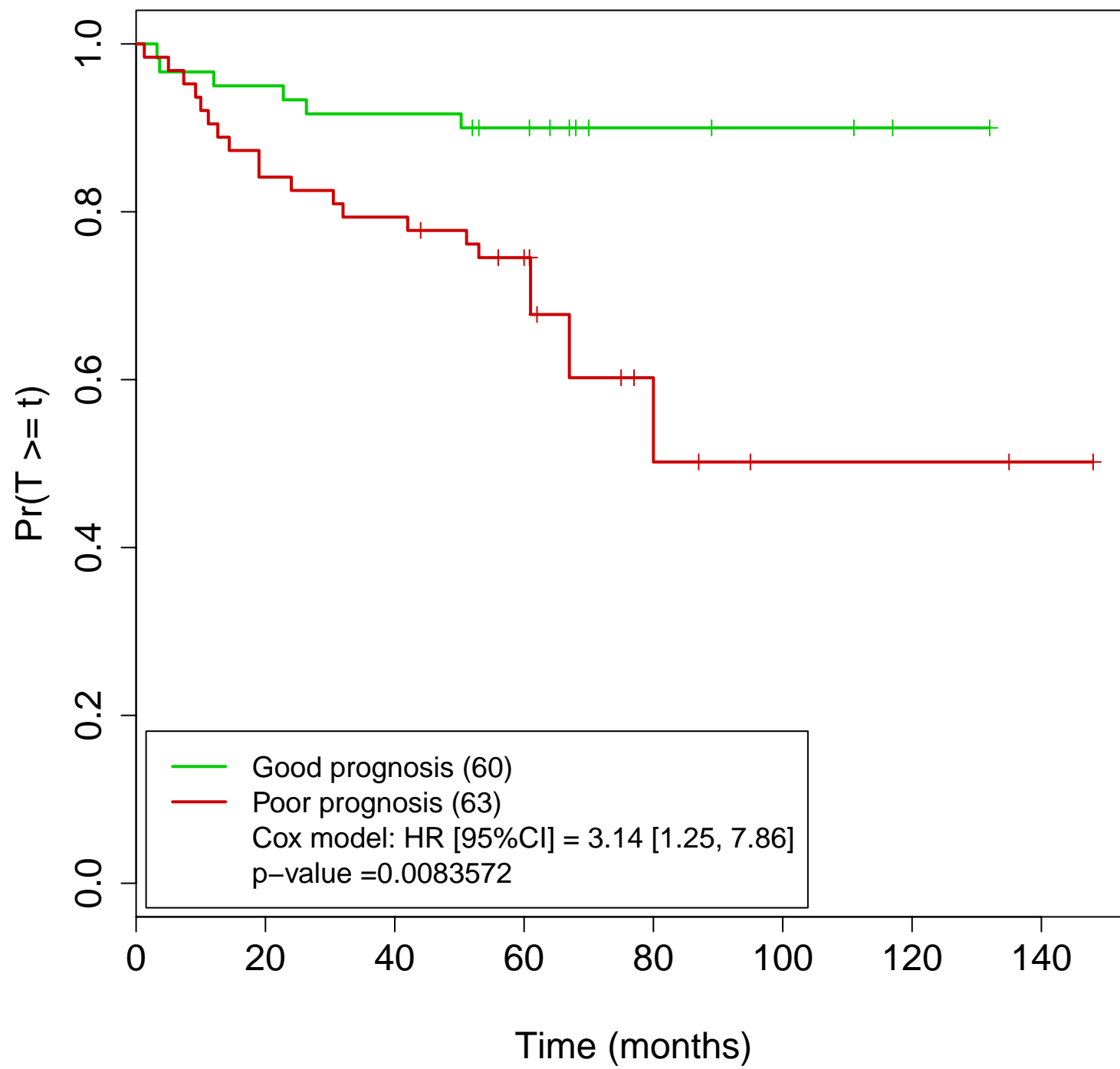

Supplement: Figure S7 — Example of outcome association in stage II samples using disease free survival information: Kaplan-Meier estimates for risk groups predicted by signature YM06 in GSE13294 dataset (Random Forest results). (PDF) [file pone.0048877.s007.pdf]

# Disease free survival for signature AJ08 in dataset GSE14333

## Stage III samples

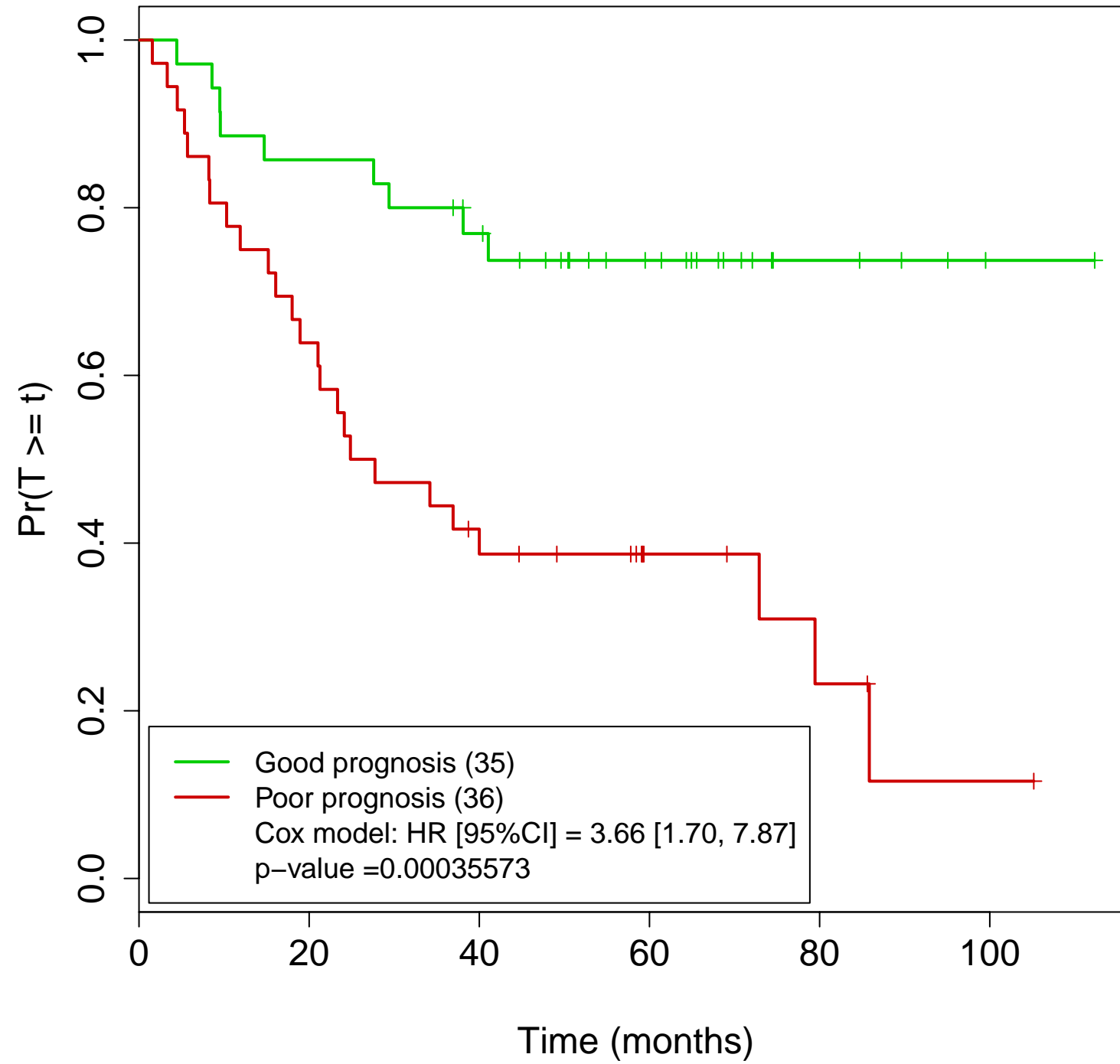

Supplement: Figure S8 — Example of outcome association in stage III samples using disease free survival information: Kaplan-Meier estimates for risk groups predicted by signature AJ08 in GSE14333 dataset (Random Forest results). (PDF) [file pone.0048877.s008.pdf]
