# Supplementary material for: Clinical Value of Prognosis Gene Expression Signatures in Colorectal Cancer: A Systematic Review
Source: PLoS One. 2012 Nov 7;7(11):e48877. doi: 10.1371/journal.pone.0048877 (PMC3492249; doi:10.1371/journal.pone.0048877)
Supplement: Table S1 — Excluded papers by eligibility criteria in the literature review. (PDF) [file pone.0048877.s009.pdf]

Supplementary Table I: Excluded papers by elegibility criteria in the literature review

STUDY	REASON FOR EXCLUSION
D'Arrigo A, Belluco C, Ambrosi A, Digito M, Esposito G, Bertola A, Fabris M, Nofrate V, Mammano E, Leon A, Nitti D, Lise M. Metastatic transcriptional pattern revealed by gene expression profiling in primary colorectal carcinoma. Int J Cancer. 2005 Jun 10;115(2):256-62.	Focused on differences between primary tumor stages (stage IV vs. non stage IV)
Fritzmann J, Morkel M, Besser D, Budczies J, Kosel F, Brembeck FH, Stein U, Fichtner I, Schlag PM, Birchmeier W. A colorectal cancer expression profile that includes transforming growth factor beta inhibitor BAMBI predicts metastatic potential. Gastroenterology. 2009 Jul;137(1):165-75. Epub 2009 Mar 26.	
Pierobon M, Calvert V, Belluco C, Garaci E, Deng J, Lise M, Nitti D, Mammano E, De Marchi F, Liotta L, Petricoin E. Multiplexed cell signaling analysis of metastatic and nonmetastatic colorectal cancer reveals COX2-EGFR signaling activation as a potential prognostic pathway biomarker. Clin Colorectal Cancer. 2009 Apr;8(2):110-7.	
Takata O, Kawamura YJ, Konishi F, Sasaki J, Kai T, Miyakura Y, Nagai H, Tsukamoto T. cDNA array analysis for prediction of hepatic metastasis of colorectal carcinoma. Surg Today. 2006;36(7):608-14.	
Spisák S, Galamb B, Sipos F, Galamb O, Wichmann B, Solymosi N, Nemes B, Molnár J, Tulassay Z, Molnár B. Applicability of antibody and mRNA expression microarrays for identifying diagnostic and progression markers of early and late stage colorectal cancer. Dis Markers. 2010;28(1):1-14.	
Zhu JS, Guo H, Song MQ, Chen GQ, Sun Q, Zhang Q. Gene profiles between non-invasive and invasive colon cancer using laser microdissection and polypeptide analysis. World J Gastroenterol. 2008 Oct 14;14(38):5887-92.	
Ki DH, Jeung HC, Park CH, Kang SH, Lee GY, Lee WS, Kim NK, Chung HC, Rha SY. Whole genome analysis for liver metastasis gene signatures in colorectal cancer. Int J Cancer. 2007 Nov 1;121(9):2005-12.	Focused on differences between primary tumor and metastases
Barrier A, Boelle PY, Lemoine A, Tse C, Brault D, Chiappini F, Lacaine F, Houry S, Huguier M, Flahault A, Dudoit S. Gene expression profiling of nonneoplastic mucosa may predict clinical outcome of colon cancer patients. Dis Colon Rectum. 2005 Dec;48(12):2238-48.	Based on normal tissue from colorectal patients
Barrier A, Roser F, Boëlle PY, Franc B, Tse C, Brault D, Lacaine F, Houry S, Callard P, Penna C, Debuire B, Flahault A, Dudoit S, Lemoine A. Prognosis of stage II colon cancer by non-neoplastic mucosa gene expression profiling. Oncogene. 2007 Apr 19;26(18):2642-8. Epub 2006 Oct 9.	
Lyall MS, Dundas SR, Curran S, Murray GI. Profiling markers of prognosis in colorectal cancer. Clin Cancer Res. 2006 Feb 15;12(4):1184-91.	Based on tissue microarrays
Zlobec I, Baker K, Terracciano LM, Lugli A. RHAMM, p21 combined phenotype identifies microsatellite instability-high colorectal cancers with a highly adverse prognosis. Clin Cancer Res. 2008 Jun 15;14(12):3798-806.	
Knösel T, Emde A, Schlüns K, Chen Y, Jürchott K, Krause M, Dietel M, Petersen I. Immunoprofiles of 11 biomarkers using tissue microarrays identify prognostic subgroups in colorectal cancer. Neoplasia. 2005 Aug;7(8):741-7.	
Hong Y, Downey T, Eu KW, Koh PK, Cheah PY. A 'metastasis-prone' signature for early-stage mismatch-repair proficient sporadic colorectal cancer patients and its implications for possible therapeutics. Clin Exp Metastasis. 2010 Feb;27(2):83-90. Epub 2010 Feb 9.	Did not provide a gene signature
Loboda A, Nebozhyn MV, Watters JW, Buser CA, Shaw PM, Huang PS, Van't Veer L, Tollenaar RA, Jackson DB, Agrawal D, Dai H, Yeatman TJ. EMT is the dominant program in human colon cancer. BMC Med Genomics. 2011 Jan 20;4:9.	
