# Supplementary material for: Clinical Value of Prognosis Gene Expression Signatures in Colorectal Cancer: A Systematic Review
Source: PLoS One. 2012 Nov 7;7(11):e48877. doi: 10.1371/journal.pone.0048877 (PMC3492249; doi:10.1371/journal.pone.0048877)
Supplement: File S1 — Details on the prognosis signatures studies systematic review containing inclusion and exclusion criteria at each step. (PDF) [file pone.0048877.s010.pdf]

PROTOCOL FOR SIGNATURES COLLECTION

Stage 1: Identification.

- Two searches on the PubMed database were done by using the following key words:
    - *(colorectal or colon) and cancer and (prognosis or prognostic or outcome or survival) and (profile or signature) and expression*
    - *("colorectal cancer" or "colon cancer") and (prognosis or prognostic or outcome or survival or metastasis or progression or recurrence) and (profile or signature or profiling) and microarray*
- Studies that reported some measure of association between prognosis in colorectal cancer and molecular characteristics at gene expression level were retained.
- All possible outcomes were considered (overall survival, disease specific survival, recurrence of any type, recurrence of some type, disease free time).

Stage 2: Screening.

- Studies that built a gene expression profile and use it to predict prognostic of CRC or, at least, to assess statistical association with prognosis were retained.
- Studies were excluded when they tried to test the prognosis value of a reduced list of candidate genes based on biology knowledge.

Stage 3: Eligibility.

- Only profiles tested on primary tumor of colorectal cancer were retained; those which were evaluated in normal tissue were excluded.
- Studies based on tissue microarray were excluded.
- Studies that focused on differences between stages or between primary tumor and metastases were excluded.
- Studies that did not provide the resulting list of genes were excluded.

Stage 4: Inclusion.

- All profiles that passed stage 3 criteria were used in qualitative and quantitative synthesis.
